# Differential Corrosion Behavior of High-Aluminum 304 Stainless Steel in Molten Nitrate Salts: The Roles of Rolling and Heat Treatment

**DOI:** 10.3390/ma18194513

**Published:** 2025-09-28

**Authors:** Weijie Tang, Kan Zhou, Zhenguo Li, Lifu Xin, Dexian Huang, Faqi Zhan, Penghui Yang, Haicun Yu, Peiqing La

**Affiliations:** 1 State Key Laboratory of Advanced Processing and Recycling of Non-Ferrous Metals, School of Materials Science and Engineering, Lanzhou University of Technology, Lanzhou 730050, China; tangweijie1116@gmail.com (W.T.); 18215161072@163.com (K.Z.); yangph@lut.edu.cn (P.Y.); yuhcyu@lut.edu.cn (H.Y.); 2 Sun Sum Technology Co. Ltd., Beijing 100102, China; lizhenguo@nmpower.com.cn (Z.L.); xinlifu@nmpower.com.cn (L.X.); 3 Sun Sum (Gansu) Technology Co. Ltd., Lanzhou 730030, China; huangdexian@nmpower.com.cn

**Keywords:** high-Al 304SS, nitrate corrosion, composite oxide film, heat treatment cold rolling

## Abstract

The high material cost has restricted the development of concentrated solar power (CSP) systems. In this study, a low-cost alternative material was developed by adding aluminum to 304 stainless steel to form a protective oxide film, thereby enhancing its corrosion resistance to molten salt. Three material variants were tested: untreated hot-rolled plates after solution treatment and cold-rolled high-aluminum 304 stainless steel (High-Al304SS) after solution treatment and annealing treatment. After all samples were immersed in a NaNO_3_-KNO_3_ mixed salt at 600 °C for 480 h, corrosion products including NaFeO_2_, CrO_2_, Mn_2_O_4_, and NiCr_2_O_4_ were formed. The phase composition was determined by XRD, and the surface and cross-section of the corrosion layer were analyzed by SEM and EDS surface and point analysis. The corrosion rate of the samples was calculated by the weight loss method. Notably, an Al_2_O_3_-Cr_2_O_3_ composite oxide film was formed on the sample surface, effectively inhibiting corrosion. The high defect density and grain boundary energy introduced by the cold-rolling process, as well as the precipitation of the second phase during annealing, accelerated the corrosion process of the samples. However, the hot-rolled samples after solution treatment exhibited excellent corrosion resistance (64.43 μm/year) and, through further process optimization, are expected to become an ideal low-cost alternative material for 347H stainless steel (23 μm/year) in CSP systems.

## 1. Introduction

Concentrated solar power (CSP) technology offers multiple advantages, including renewability, cleanliness, adjustability, reduced energy costs, and enhanced energy security, making it an efficient, environmentally friendly, and sustainable energy solution [1]. In second-generation CSP systems, nitrate salts (NaNO_3_-KNO_3_, with a mass ratio of 60–40%) are widely employed as heat storage media. Elevating the maximum operating temperature of the molten salt cycle can significantly enhance the overall efficiency of the power plant [2,3,4,5]. However, the stability and performance of structural materials at elevated temperatures remain key limiting factors for further increasing the operational temperature, particularly in thermal energy storage (TES) systems and receiver components. Currently, the operational temperature of commercial nitrate salts is typically restricted to below 565 °C [6]. At the same time, heat transfer fluids used in high-temperature environments must meet conventional mechanical performance requirements, as elevated temperatures can intensify corrosion and thermal stress, thereby compromising mechanical strength and long-term durability [7,8,9,10,11]. Austenitic stainless steels are extensively used in core high-temperature components such as receivers and high-temperature storage systems due to their low cost, high strength, excellent high-temperature resistance, oxidation resistance, corrosion resistance, and creep strength [5,12]. Among them, the Japanese-developed TP347H austenitic heat-resistant steel, benefiting from its specialized thermomechanical processing that results in finer grain sizes, exhibits superior resistance to high-temperature steam oxidation, corrosion, and favorable creep strength. As a result, it has been adopted as a structural material for certain high-temperature components in CSP systems. However, its high cost limits its widespread application. In contrast, conventional 304 austenitic stainless steel is more cost-effective but fails to meet the corrosion resistance requirements under molten salt conditions at 600 °C in medium-to-low temperature trough-type CSP systems (with an operational temperature of approximately 560 °C), posing significant operational risks and application constraints. Therefore, it is imperative to enhance its high-temperature corrosion resistance.

Research indicates that the introduction of aluminum (Al) into austenitic alloys facilitates the formation of an oxide film predominantly composed of Al_2_O_3_, which exhibits superior thermodynamic stability and enhanced high-temperature oxidation resistance compared to the conventional Cr_2_O_3_-based oxide film. Furthermore, Al addition during the solution annealing of austenitic stainless steel not only promotes grain refinement but also enhances the dislocation pinning effect, thereby effectively suppressing grain growth [13]. In ferritic stainless steels, Al incorporation significantly increases the proportion of equiaxed grains, stabilizes the ferrite phase, and reduces the recrystallization temperature, accompanied by an increase in both the content and size of Al_2_O_3_ precipitates [14,15,16,17,18]. Moreover, grain refinement induced by hot rolling has been shown to improve the localized corrosion resistance of 304 stainless steel [19,20]. Austenitic stainless steels demonstrate excellent corrosion resistance due to the spontaneous formation of a protective passive film on their surface. However, in environments containing trace halides, this passive film may undergo localized breakdown, initiating pitting corrosion. Such localized corrosion is not only detrimental on its own but can also act as a precursor to stress corrosion cracking (SCC), potentially compromising the structural integrity of the material [21,22,23,24,25]. Existing studies have demonstrated that localized corrosion is often associated with microstructural heterogeneities, such as non-metallic inclusions, intermetallic phases, ferrite regions, strain-induced martensite, and grain boundaries. Solid solution treatment can effectively homogenize the alloy composition. Additionally, it has been reported that during cold rolling and subsequent annealing of 304 austenitic stainless steel, extensive martensitic reverse transformation occurs. These transformation products can serve as preferential nucleation sites for recrystallization, thereby promoting grain refinement and enhancing the overall corrosion resistance of the material [26,27,28,29]. In the Austenite cold rolling experiments [30,31,32], cold rolling significantly reduced the corrosion rate of the material and decreased the grain size while increasing the low-angle grain boundaries (LAGBs). LAGBs have a lower energy level, which makes them more stable in corrosive environments and can effectively restrict the rapid diffusion of corrosive ions. In contrast, high-angle grain boundaries (HAGBs) are usually regarded as fast diffusion channels and are more susceptible to corrosion. The cold-rolled martensitic can be re-crystallized and grown through annealing or solution treatment, releasing the energy stored due to the martensitic phase transition, thereby preventing stress corrosion [33,34,35,36,37].

In previous studies of our research group, aluminum (Al) was introduced into the 625 alloy. The addition of Al promotes the formation of a dense Al_2_O_3_ layer, effectively blocking the penetration of molten salt. Meanwhile, the introduction of magnesium (Mg) into the chloride salt facilitates the formation of MgCr_2_O_4_ with chromium (Cr), reducing the dissolution loss of Cr. It also reacts with impurities such as MgOHCl, HCl, and Cl_2_ to generate MgO, thereby reducing corrosive gases [38,39]. In this study, the grain size of the High-Al 304 hot-rolled plate was reduced by cold rolling, making the small-angle grain boundaries dominant. In the hot-rolled sheet, the proportion of small-angle grain boundaries reached 61%. After cold rolling, the proportion of small-angle grain boundaries increased to 79% and became dominant. Finally, after solution treatment and annealing, the grain structure became more uniform, and the residual stress inside the material was also reduced.

## 2. Experimental Section

### 2.1. Materials

The chemical compositions of 304 SS and High-Al 304SS are shown in Table 1. The High-Al 304SS (with a thickness of 6 mm) used in this experiment was produced and processed by a steel company.

### 2.2. Experimental Procedure

#### 2.2.1. Treatment Processes of Steels

Hot rolled High-Al 304SS followed by solution treatment (HR-Solution): Solution treatment and annealing experiments were conducted using an SX-G16103 muffle furnace (Shanghai Qianjun Scientific Instrument Co., Ltd., Shanghai, China). The samples were heated from room temperature to the target solution treatment temperature (1050 °C), held for 30 min, and then rapidly water-quenched to room temperature.

Cold-rolled High-Al 304SS was then subjected to solution treatment (CR-Solution): The hot-rolled stainless steel plates cut by electrical discharge were cold-rolled at room temperature using a two-roll hot/cold rolling mill (ZK-WS2A4; Zhikuo Precision Machinery Co., Ltd., Dongguan, China). During the rolling process, the reduction per pass was controlled to be less than 0.2 mm, and the rolling speed was 0.4 m/min. The mill speed was controlled at 15 rpm, resulting in a 90% deformation rate of 304 thin plates. The treatment method of the cold-rolled plates was the same as that of the hot-rolled (solution-treated) plates. Meanwhile, the rolling parameters of hot rolling, except for the temperature (1200 °C), were the same as those of cold rolling.

Cold rolled High-Al 304SS followed by annealing (CR-Annealing): Cold rolling was conducted in the same manner as described for the CR-Solution samples, followed by annealing in a resistance furnace at 800 °C for 60 min.

#### 2.2.2. Corrosion Experiment

Several sample blocks with dimensions of 12 × 12 × 0.68 m^3^ were cut from High-Al 304SS thin plates (annealed and solution-treated, CR90%) and hot-rolled plates (solution-treated) using an electric spark wire-cutting machine (FZ-440SF Wire-Cut EDM Machine, Jiangsu Fangzheng CNC Machine Tool Co., Ltd., Taizhou, China). The surfaces of the specimens were ground with 120–2000 grit SiC sandpaper. The dimensions of each specimen were measured sequentially using a vernier caliper and recorded accordingly. The mass of each specimen was determined using an electronic balance (Accuracy of 0.0001 g). The experimental binary nitrate salts (NaNO_3_-KNO_3_, 60:40 wt%) was homogenized by mortar grinding, oven-dried at 120 °C for 12 h, and stored in a desiccator until use.

The corrosion test was carried out under an air atmosphere using the static immersion method, with a binary molten nitrate salt as the corrosive medium. The test temperature was maintained at 600 °C, and the total exposure duration was 480 h. During the test, samples were retrieved for analysis at six predetermined time intervals: 72, 144, 216, 288, 384, and 480 h. In addition, four parallel samples were prepared for each time point to minimize the influence of random experimental errors. When the experiment reaches the predetermined corrosion time point, the crucible is removed and allowed to cool naturally in air. The corroded samples, together with any residual salt, are then placed in distilled water and ultrasonically cleaned for 10 min. Subsequently, the samples are ultrasonically cleaned in anhydrous ethanol for 5 min, following the cleaning procedure for corrosion sample outlined in the ASTM G1-03; Standard Practice for Preparation, Cleaning and Evaluation of Corrosion Test Specimens. ASTM International: West Conshohocken, PA, USA, 2003 [40]. The cleaning solution is prepared with a composition of HNO_3_:HF:distilled water in a volumetric ratio of 5:1:44. The corroded samples are then immersed in the prepared cleaning solution and subjected to ultrasonic cleaning for a duration of 30 s.

The corrosion rate (*V*) is determined following the procedures outlined in the ASTM G1-03 [40] standard. The mass difference in the samples before and after the corrosion test is measured and analyzed. Based on this data, the mass change per unit area (mg/cm^2^) and the annual metal thickness loss (μm/year) are calculated by Formulas (1) and (2).

(1) $$\frac{∆m}{S_{0}}=\frac{m_{t}-m_{0}}{S_{0}}$$

where *m*_t_ is the mass of the sample after corrosion for a certain period of time, in grams; *m*_0_ is the initial mass of the sample, in grams; *S*_0_ is the initial surface area of the sample, in cm^2^.

(2) $$V=\frac{K\;∆m}{S_{0}\;t\;\rho }$$

where *K* is a constant equal to 8.76 × 10^7^ μm/year; Δ*m* is the mass loss of the sample during the corrosion process, in grams; *S_0_* is the initial surface area of the sample, in square centimeters; *t* is the corrosion time of the sample, and *ρ* is the density of the sample, in grams per cubic centimeter (the density of the sample *ρ* = 7.8 g/cm^3^).

### 2.3. Microstructural Characterization

X-ray Diffraction (XRD) Analysis: The phase composition of the corrosion products was investigated using a DX-2800 X-ray diffractometer (DX-2800, Dandong Haoyuan Instrument Co., Ltd., Dandong, China) by scanning the surface of the corroded samples. The X-ray diffraction (XRD) scanning parameters are set as follows: the filter is Cu Kα, the monochromator λ = 1.5406 nm, the 2θ diffraction angle range is from 20 to 120 degrees, the scanning speed is 10 degrees per minute, the accelerating voltage is 40 kV, and the current is 150 mA. Scanning Electron Microscope (SEM) Analysis: The surface corrosion layer and cross-sectional morphology of the corroded samples were examined using a Guoyi Quantum SEM5000 field emission electron microscope (SEM5000, Guoyi Quantum Co., Ltd., Hefei, China) at an accelerating voltage of 20 kV. In order to determine the elemental composition of the corrosion products, energy dispersive spectrometry (EDS) analysis was conducted using the microscope’s built-in EDS system (SEM5000, Guoyi Quantum Co., Ltd., Hefei, China), incorporating point, line, and area scanning modes.

## 3. Results

### 3.1. Molten Salt Corrosion Resistance Performance

**Figure 1:**
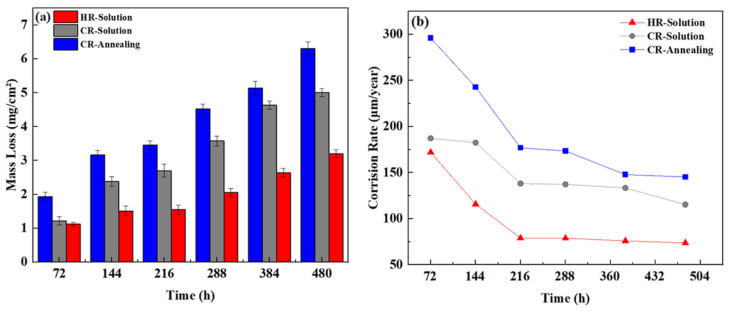
(**a**) Mass change per unit area of High-Al 304SS after corrosion for different periods of time; (**b**) corrosion rate curve of High-Al 304SS.

Using Formulas (1) and (2), the mass change per unit area and corrosion rate values of High-Al 304SS subjected to different processing methods were calculated after corrosion in a binary molten nitrate salt at 600 °C for varying durations. The resulting values were plotted as curves, as shown in Figure 1a (mass change per unit surface area) and Figure 1b (corrosion rate curve). As shown in Figure 1a, the corrosion rates of High-Al 304SS cold-rolled sheets and hot-rolled sheets treated by solid solution treatment are lower than those of annealed samples. The quality change trends of two different heat-treated High-Al 304SS cold-rolled sheets are basically the same and can be divided into two stages. Before 216 h, the unit area mass changes rapidly. At 216 h, the unit area mass increase in the CR-Solution is 2.70 mg/cm^2^, while that of the annealed cold-rolled sheet is 3.46 mg/cm^2^. After 216 h, the change in unit area mass of the samples slows down. In Figure 1b, the corrosion rate of the annealed cold-rolled sheet at 72 h is 295.55 μm/year, significantly higher than that of the CR-Solution at 187.21 μm/year. The corrosion rate of CR-Annealing showed a significant decrease before 216 h, with the rate of decrease slowing after 216 h and eventually stabilizing after 384 h, reaching a final corrosion rate of 145.2 μm/year. Similarly, the corrosion rate of CR-Solution stabilized after 216 h and reached its lowest point at 480 h, with a corrosion rate of 115.26 μm/year. Similarly, as shown in Figure 1b, the corrosion rate of hot-rolled High-Al 304SS is relatively high at 72 h, reaching 172.01 μm/year. As the corrosion time increases, the corrosion rate decreases significantly before 216 h. After 216 h, the corrosion rate gradually stabilizes, and by 480 h, the corrosion rate decreases to 64.43 μm/year. Based on the corrosion rate curve, a protective oxide layer forms after 216 h, slowing down the corrosion of the base material by the molten salt. Therefore, the corrosion rate becomes more stable after 216 h. According to the corrosion resistance standards for metals, the corrosion rate of this HR-Solution in molten nitrate salts environment is as low as 64.43 μm/year, meeting the service requirements for CSP.

### 3.2. Phase Analysis of Corrosion Products

**Figure 2:**
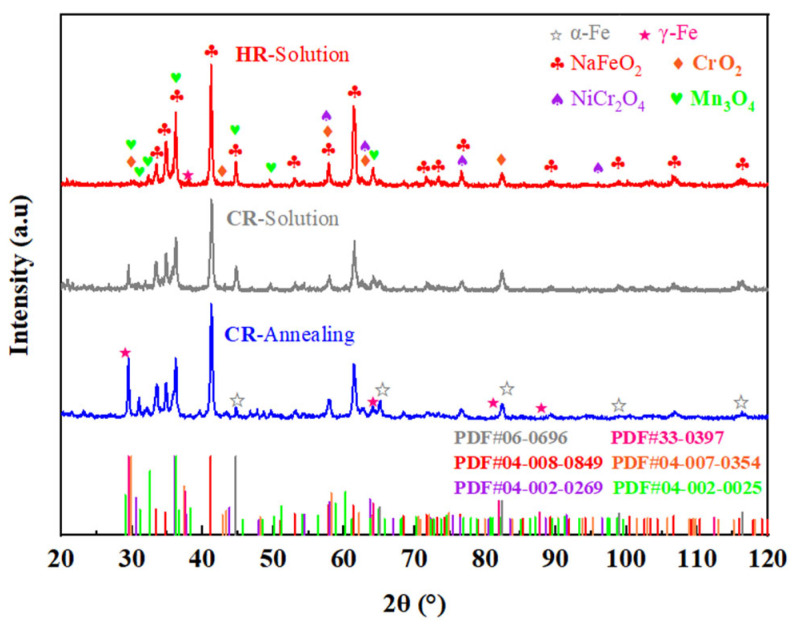
XRD patterns of the surface of High-Al 304SS samples after 480 h of corrosion under different conditions.

The XRD characterization results in Figure 2 reveal the surface phase composition of High-Al 304SS after 480 h exposure to binary molten nitrate salts (60% NaNO_3_-40% KNO_3_) at 600 °C under various corrosion conditions. As can be seen from Figure 2, the corrosion products are primarily NaFeO_2_ (PDF #04-008-0849). Additionally, peaks corresponding to NiCr_2_O_4_ (PDF #04-002-0269) are observed in the figure. Furthermore, oxides of manganese and chromium, namely Mn_3_O_4_ (PDF #04-002-0025) and CrO_2_ (PDF #04-007-0354), can also be detected. Semi-quantitative analysis of the obtained XRD data is presented in Table 2. As shown in the table, NaFeO_2_ is the primary corrosion product, accounting for 54.0% of the total weight, followed by Mn_3_O_4_ at 19.6%, and NiCr_2_O_4_ at 13.3%. The phase analysis of the corrosion products through XRD revealed that the austenite composition structure was particularly prominent in the CR-Annealing and CR-Solution samples, and its diffraction intensity gradually decreased from CR-Annealing, CR-Solution to HR-Solution. In contrast, the ferrite phase could be clearly detected in all three types of corrosion products.

### 3.3. The Microstructure of the Surface Before Corrosion

**Figure 3:**
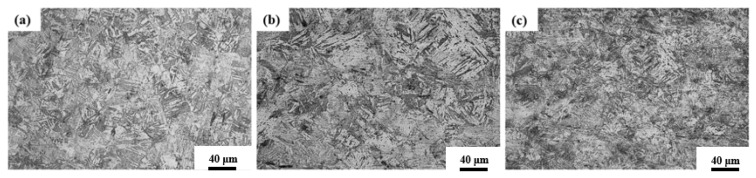
OM diagrams of samples before corrosion: (**a**) CR-Annealing, (**b**) HR-Solution and (**c**) CR-Solution.

Figure 3 presents the crystal orientation diagrams of CR-Annealing (a), CR-Solution (b), and HR-Solution (c) after processing. As shown in Figure 3, sample (a) exhibits a typical lamellar martensite microstructure (Figure S1), whereas the solution-treated samples display a two-phase structure composed of ferrite and austenite. The austenite undergoes martensitic transformation during cold rolling, resulting in the formation of a martensitic structure. Following solution treatment, this martensitic structure undergoes recrystallization and grain growth during the annealing process (Figure S2).

### 3.4. The Microstructure and Composition of the Corroded Surface

**Figure 4:**
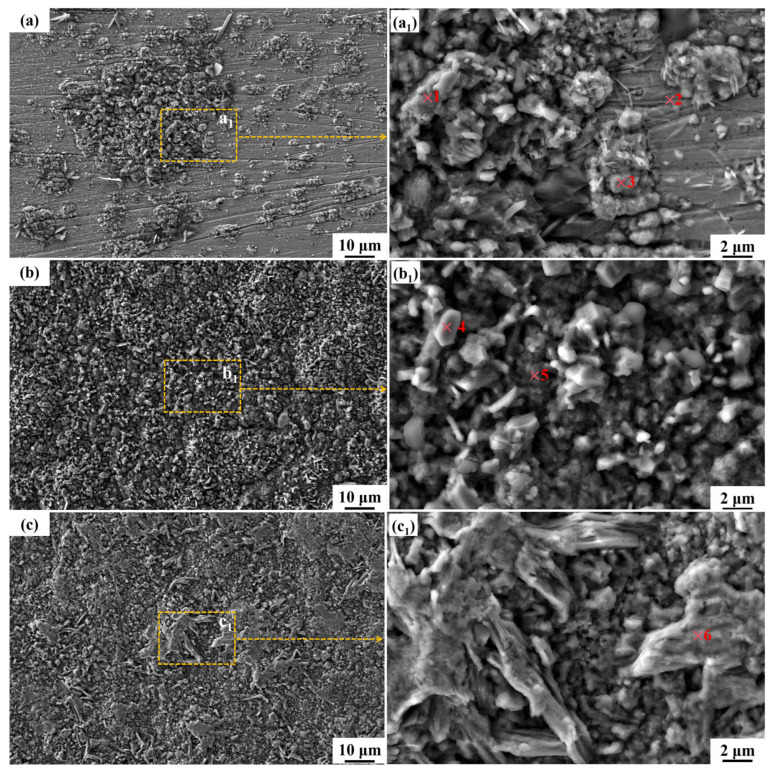
SEM images of the surface morphology of HR-Solution sample after corrosion in molten salt for different durations: (**a**) 72 h; (**b**) 216 h; (**c**) 480 h. (**a_1_**), (**b_1_**), and (**c_1_**) are respectively the magnified micrographs of the yellow-marked areas in the main figure. The numbers highlighted in red were selected for EDS point-scanning.

After 30 min of solution treatment at 1050 °C, the surface morphology of HR-Solution after corrosion in binary nitrate salts at 600 °C for different periods of time is shown in Figure 4. From the low-magnification SEM image, it can be seen that the surface of the sample becomes rough after corrosion, with corrosion products distributed on the substrate in different shapes and sizes, and some areas completely covered. During the early stages of corrosion, the corrosion products do not completely cover the substrate, leaving part of the substrate exposed. As the corrosion time increases, the corrosion products gradually accumulate, completely covering the substrate. The morphology of corrosion products also changes with increasing corrosion time. After 72 h of corrosion, the corrosion products on the sample surface are irregular cubes of varying sizes. ${\mathrm{NO}}_{3}^{-}$ in the molten salt produces O^2−^, which reacts with the substrate to form Fe_3_O_4_. After oxidation, Fe_2_O_3_ is formed, which then combines with Na_2_O in the molten salt to form NaFeO_2_ particles. At this point, the oxide film has not fully covered the surface, and the corrosion rate reaches 172.01 μm/year; as the corrosion time is extended to 216 h, the number of corrosion products increases, taking on smaller, irregular shapes. As the number of NaFeO_2_ particles increases, grain boundary diffusion promotes particle fusion, forming a continuous layer; Cr and Al begin to migrate toward the surface, forming a Cr_2_O_3_-Al_2_O_3_ transition layer in the intergranular spaces, reducing the corrosion rate to approximately 90.5 μm/year; when the corrosion time is further extended to 480 h, the size of the polyhedral corrosion products increases, with some transforming into a fully developed, continuous, and densely packed state with smooth edges, while other areas exhibit corrosion products resembling scales in shape. The NaFeO_2_ layer interacts synergistically with the inner Cr_2_O_3_-Al_2_O_3_ composite film to hinder ${\mathrm{NO}}_{3}^{-}$ penetration, stabilizing the corrosion rate at 64.43 μm/year. Comprehensive observations reveal that as corrosion time increases, corrosion product sizes gradually grow larger, exhibiting improved continuity and density, and providing better coverage of the substrate.

To determine whether changes in the crystal shape and size of surface corrosion products are accompanied by changes in composition, EDS point analysis was performed on corrosion products with different morphologies on the surfaces of samples corroded for different lengths of time, as shown in Table 3. Based on the atomic fraction ratio, high O and Na contents (O ≈ 50–55%, Na ≈ 15–25%) were determined, which are consistent with the chemical composition of NaFeO_2_. The Fe content is in a stoichiometric ratio with Na (Na/Fe ≈ 1), and there is no high proportion of matrix Fe (the matrix Fe content is usually > 60%). At point 1, the Na/Fe ratio is 1.09, which is close to the theoretical value of NaFeO_2_ (1:1). The O content of 53.36% aligns with the oxygen proportion in NaFeO_2_ (50%), indicating that NaFeO_2_ is the dominant corrosion product. At this point, Al is 1.03%, and it resides in the outer layer of the corrosion product. The inner Al_2_O_3_ barrier layer (Al > 4%) is situated at a deeper level, effectively impeding the penetration of molten salt. At point 2, the Na/Fe ratio is 0.06, and Fe reaches 41.86%, which is significantly higher than the theoretical value for NaFeO_2_. This suggests that the region corresponds to an exposed matrix area, covered only by a thin layer of Fe_2_O_3_, which has not fully reacted with Na^+^. The highest Al (15.16%) and Cr (3.03%) concentrations were observed across the entire surface at the matrix-corrosion product interface, where an initial Al_2_O_3_-Cr_2_O_3_ composite oxide film forms, effectively inhibiting molten salt penetration. However, due to incomplete coverage of the product layer, the corrosion rate reached 172.01 μm/year. NiCr_2_O_4_ is also produced at the same time. At point 3, the Na/Fe ratio is 0.6, which falls between the values characteristic of Fe_2_O_3_ and NaFeO_2_, with an O content of 62.79%. This indicates that Fe_2_O_3_ is the dominant corrosion product (excess product layer), while NaFeO_2_ begins to form locally. At point 4, the Na/Fe ratio is 1.05, close to the theoretical value for NaFeO_2_, and the O content is 52.86%, indicating that NaFeO_2_ is the primary product (fusion layer). Si (0.16%) fills intergranular gaps, thereby enhancing the compactness of the corrosion layer. Al is 1.53%, lower than at point 3, indicating that as the product layer thickens, the diffusion resistance of aluminum increases. Consequently, the protective function of the outer layer is primarily attributed to NaFeO_2_, while the barrier effect of the inner Al_2_O_3_ layer diminishes. At point 5, the Na/Fe ratio is 1.63, indicating a significantly excessive sodium content, which suggests the potential formation of Na_2_O and NaFeO_2_. The O content of 52.74% indicates that the corrosion product has undergone dehydration and crystallization. Al is 1.52%, which is comparable to the value at point 4, indicating that the outer layer product is predominantly NaFeO_2_ (mature corrosion layer). At point 6, Al is 0.16%, the lowest value in the entire dataset, indicating that a mature corrosion layer has formed, with NaFeO_2_ as the dominant outer layer product and an Al_2_O_3_ barrier layer formed in the inner layer that no longer allows outward diffusion.

**Figure 5:**
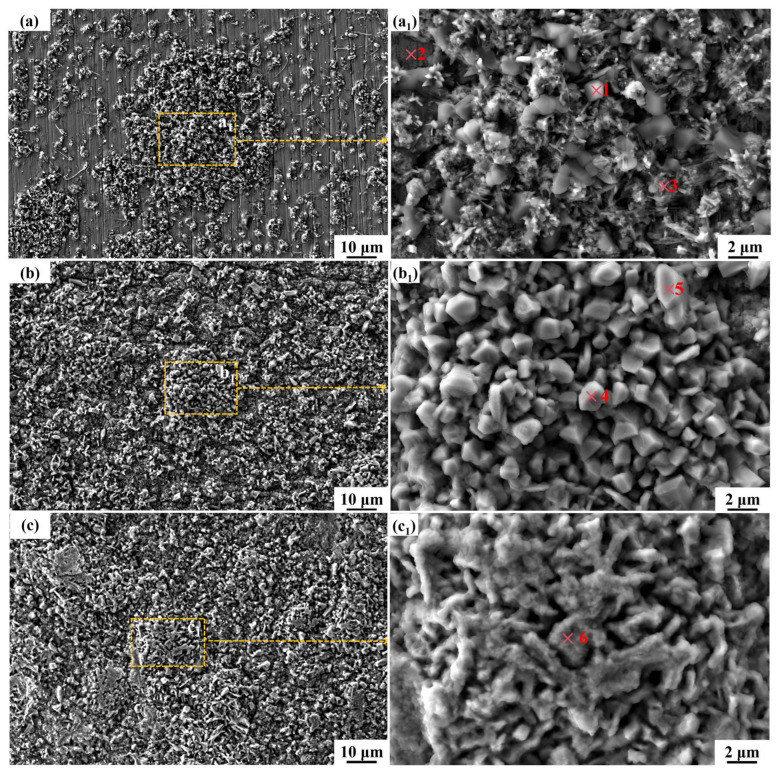
SEM micrographs of the surface morphology of CR-Solution sample after corrosion in molten salt for varying durations: (**a**) 72 h; (**b**) 216 h; (**c**) 480 h. (**a_1_**), (**b_1_**), and (**c_1_**) are respectively the magnified micrographs of the yellow-marked areas in the main figure. The numbers highlighted in red were selected for EDS point-scanning.

The SEM micrographs of the CR-Solution after varying corrosion durations in a binary molten nitrate salts environment are presented in Figure 5. As observed in the images, during the early stages of corrosion, the corrosion products do not fully cover the substrate surface. However, with increasing corrosion time, the continuity and compactness of the corrosion layer progressively improve. The corrosion products are predominantly polygonal in morphology. As the exposure time increases, the size of these polygonal structures gradually enlarges, and some evolve into a more continuous and smooth-edged morphology. Based on the morphological observations, it can be inferred that the corrosion products progressively coalesce and interconnect over time, leading to a gradual expansion of individual corrosion zones. Furthermore, the edges of the corrosion products transition from a sharp to a smoother appearance, and the boundaries between adjacent corrosion regions gradually merge, ultimately forming a more continuous and densely packed corrosion layer.

EDS point analysis was conducted on the corrosion products at different locations in Figure 5, and the results are summarized in Table 4. These findings are consistent with the EDS point scan analysis of the corrosion-affected surface of CR-Solution. The corrosion products on the surface of this group of samples are primarily iron-rich oxides, with the main constituent elements being Fe, O, and Na. Corrosion time has a certain influence on the morphology and size of the corrosion products, but their chemical composition remains unchanged. Comparison with the XRD results indicates that the primary corrosion product is NaFeO_2_. Using the same analytical method as described in Table 3, Table 4 shows that a composite oxide film composed of Al_2_O_3_ and Cr_2_O_3_ is attached to the matrix at point 2, with NiCr_2_O_4_ also present; however, Al_2_O_3_ is the dominant component. Points 1, 4, 5, and 6 all correspond to NaFeO_2_, while point 3 corresponds to an iron oxide.

**Figure 6:**
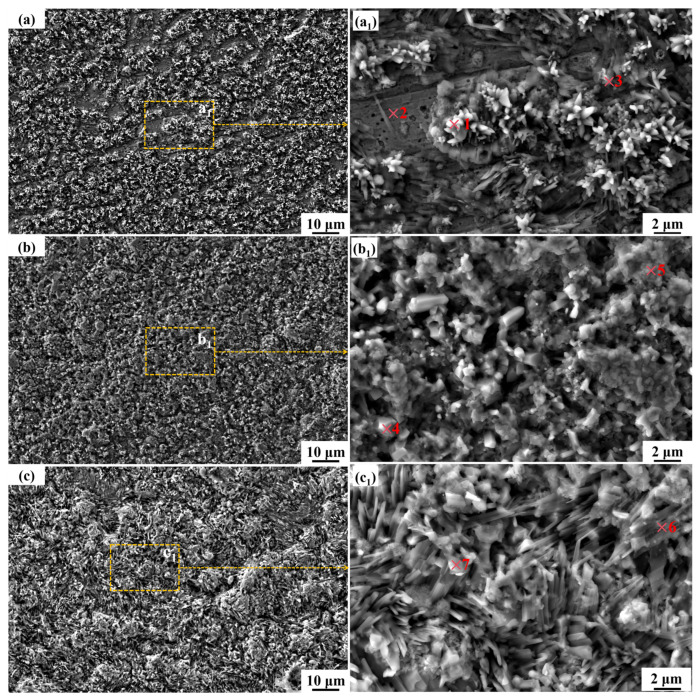
SEM micrographs of the surface morphology of CR-Annealing after corrosion in molten salt for varying durations: (**a**) 72 h; (**b**) 216 h; (**c**) 480 h. (**a_1_**), (**b_1_**), and (**c_1_**) are respectively the magnified micrographs of the yellow-marked areas in the main figure. The numbers highlighted in red were selected for EDS point-scanning.

The SEM morphology of CR-Annealing sample after corrosion for various durations in a binary nitrate molten salt is presented in Figure 6. It can be observed that in the early stage of corrosion, the corrosion products are sparsely distributed on the substrate surface and do not fully cover the base material. As the corrosion time increases, the corrosion products gradually develop into a continuous layer that completely covers the substrate. The corrosion products are predominantly polyhedral in shape, with a small number of strip-like structures interspersed among them. With further prolongation of the corrosion time, the size of the corrosion products increases gradually, and their structure becomes more continuous and compact, with the edges of some polyhedral structures becoming smoother. This evolution trend is generally consistent with the morphological and dimensional changes observed in the corrosion products formed on the surfaces of High-Al 304SS hot-rolled plates and solution-treated High-Al 304SS thin sheets. For all three groups of samples, the size of the surface corrosion products increases progressively with increasing corrosion time. Additionally, the morphology of the corrosion products becomes smoother and more continuous over time, and the corrosion layer gradually becomes more compact. Consequently, the corrosion layer can more effectively inhibit further attack by the molten salt, which is also a key factor contributing to the gradual decrease and eventual stabilization of the corrosion rate with prolonged exposure time.

The EDS analysis was performed on corrosion products of different morphologies and sizes on the surfaces of specimens with different corrosion durations shown in Figure 6. The results are shown in Table 5. From the EDS point scan results, it can be seen that as the corrosion time increases, the morphology and size of the corrosion products undergo different changes, but their main elements remain Fe, O, and Na. Combined with XRD, it can be concluded that the corrosion products are mainly NaFeO_2_. The results are similar to those of the previous two corrosion test samples, indicating that during the corrosion process, the corrosion products formed on the surface of the High-Al 304SS test samples are primarily NaFeO_2_, and the composition of the corrosion products is not affected by differences in the pre-corrosion heat treatment process or cold rolling process. Points 1, 3, 4, 5, 6, and 7 are all corrosion products of NaFeO_2_, while point 2 is a composite oxide film of Al_2_O_3_-Cr_2_O_3_ and NiCr_2_O_4_ formed on the matrix at the initial stage of corrosion. However, during the annealing process, the contents of the two oxide films in the composite oxide film are basically equivalent.

**Figure 7:**
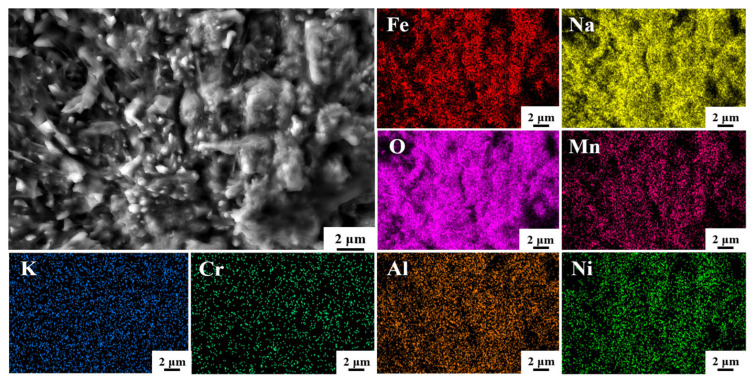
SEM morphology and EDS mapping of HR-Solution sample after 480 h molten salt corrosion.

The surface morphology and EDS elemental analysis results of the sample surface of HR-Solution after 480 h of corrosion in a binary molten nitrate salt at 600 °C are shown in Figure 7. From the figure, it can be seen that the surface mainly consists of strip-shaped corrosion products, interspersed with polyhedral-shaped corrosion products. The strip-shaped products are mainly composed of Fe, Na, and O elements, and the corrosion products are mainly NaFeO_2_. Some areas are enriched with Mn elements, forming Mn_2_O_4_ oxides. The overlap of Al and O elements, as well as Cr and O elements, confirms the conclusion that Al_2_O_3_ and Cr_2_O_3_ thin films are formed, while Cr elements are uniformly distributed in both products in the form of Cr_2_O_3_ and NiCr_2_O_4_.

**Figure 8:**
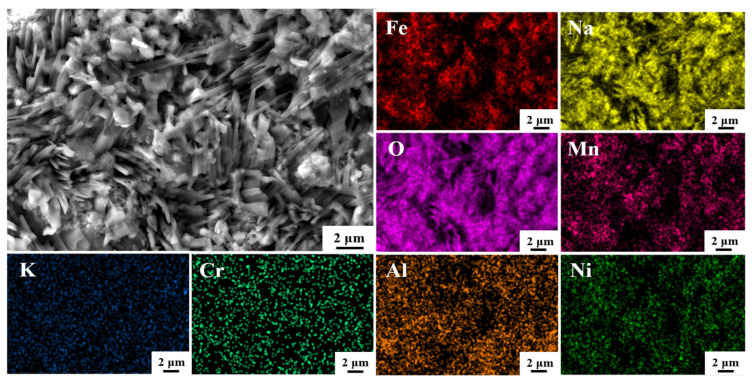
SEM morphology and EDS mapping of CR-Annealing sample after 480 h molten salt corrosion.

The surface morphology of corrosion products formed on CR-Annealing is shown in Figure 8, along with the EDS elemental analysis results, following 480 h of corrosion in a binary molten nitrate salt. In this figure, the surface corrosion products are predominantly polyhedral in shape, with some elongated structures interspersed. According to the EDS element distribution, the main constituent elements of the corrosion products are Fe, Na, and O. The elongated corrosion products exhibit Mn enrichment, and XRD analysis confirms that NaFeO_2_ is the primary corrosion product. The presence of Mn enrichment further suggests the formation of Mn-containing oxides such as Mn_3_O_4_. Cr is uniformly distributed across both types of corrosion products, indicating the formation of an Al_2_O_3_-Cr_2_O_3_ composite oxide film and NiCr_2_O_4_.

**Figure 9:**
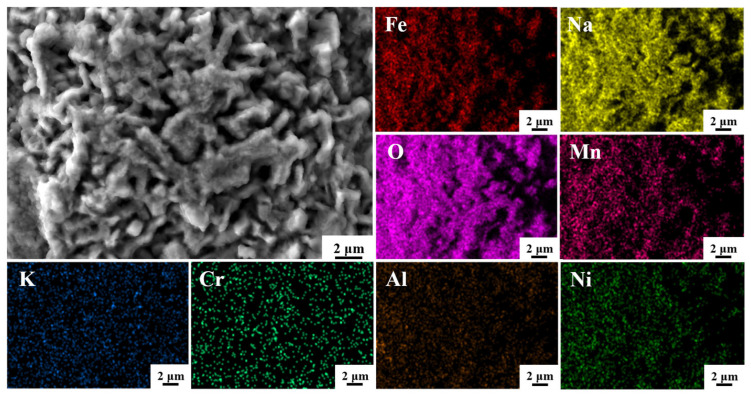
SEM morphology and EDS mapping of CR-Solution sample after 480 h molten salt corrosion.

The surface morphology and EDS elemental analysis results of CR-Solution sample after 480 h of corrosion in a binary nitrate molten salt are presented in Figure 9. Compared with the corrosion product morphology of the annealed thin plate under the same corrosion conditions, the solution-treated thin plate developed a more compact surface oxide layer after corrosion, and its polyhedral corrosion products exhibited more rounded shapes, improved continuity, and smoother edges. The main constituent elements of the corrosion products remained Fe, Na, and O, with NaFeO_2_ as the dominant phase. Additionally, an Al_2_O_3_-Cr_2_O_3_ composite oxide film and NiCr_2_O_4_ were simultaneously formed.

### 3.5. The Microstructure and Composition of the Corroded Cross-Section

To ensure that the elements were not affected by segregation or diffusion caused by previous heat treatment and rolling processes before the corrosion test, cross-sectional observations and EDS analyses were conducted on three samples before corrosion. The results are shown in Figures S3–S5, which correspond to the EDS spectra of the CR-Annealing, CR-Solution, and HR-Solution samples, respectively. As shown in Figures S3 and S4, Fe, Ni, Cr, Mn, Si, and S elements are uniformly distributed in the matrix. Additionally, the presence of Al and C elements from the workbench led to the observed layering phenomenon. In Figure S5, the inclined cross-section of the corrosion layer caused the scanning results of the matrix to be inclined; however, the consistent layering pattern is still obvious. These observations indicate that no significant element segregation occurred during the rolling or heat treatment processes before corrosion.

**Figure 10:**
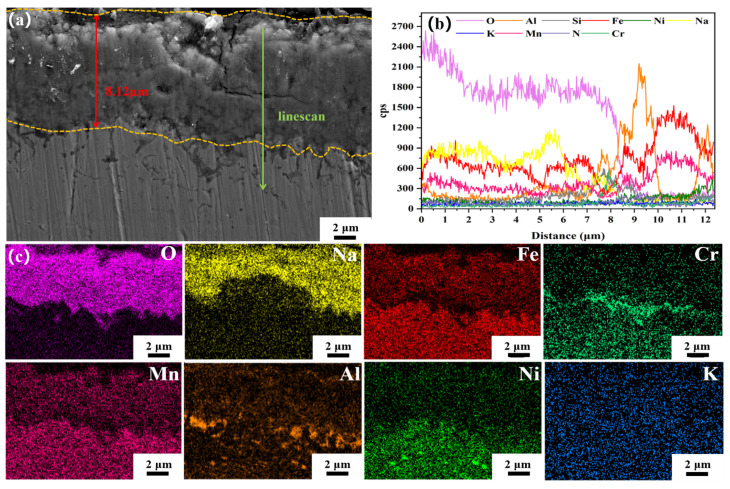
(**a**) SEM images of the cross-section of the corrosion layer of HR-Solution sample after 480 h of corrosion in molten salt, (**b**) EDS line scan analysis, (**c**) EDS area scan analysis.

The SEM and EDS analyses of the corrosion layer cross-section of the HR-Solution sample after 480 h of corrosion in a binary molten nitrate salt are shown in Figure 10. At this point, the corrosion layer thickness was 8.12 μm (±0.10 μm). The corrosion products were mainly composed of Fe, Na, and O elements, which were identified as NaFeO_2_ based on XRD results. There was an enrichment of Cr and Al elements in the area of the corrosion layer close to the matrix, and the inner side of the corrosion layer close to the matrix showed a dark image. Based on the line scan results, it was determined that nitrate had entered this area. This phenomenon indicates that the corrosion layer is not completely compact but contains some voids. During corrosion, these voids provide a pathway for binary molten nitrate salts, resulting in a darker brightness in this area.

**Figure 11:**
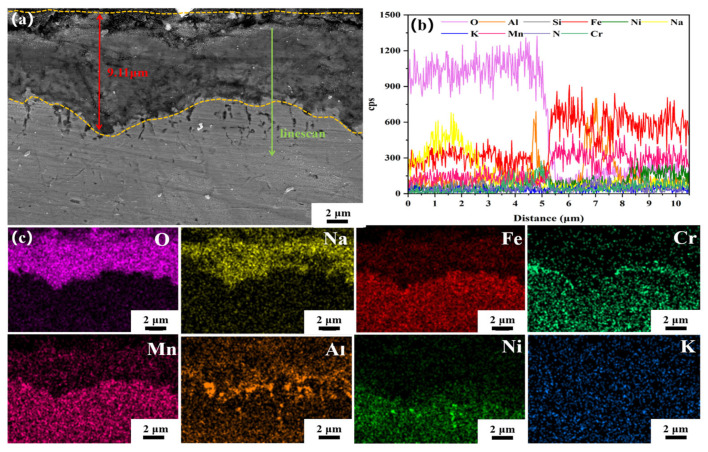
(**a**) SEM images of the cross-section of the corrosion layer of CR-Annealing sample after 480 h of corrosion in molten salt, (**b**) EDS line scan analysis, (**c**) EDS area scan analysis.

The SEM microstructure and EDS analysis of the cross-sectional corrosion layer formed on CR-Annealing sample are presented in Figure 11, which was annealed at 800 °C and subsequently corroded in a binary nitrate molten salt at 600 °C for 480 h. The corrosion layer exhibits an average thickness of approximately 9.11 μm (± 0.10 μm). The EDS line and area scanning analyses reveal that the primary elemental constituents of the corrosion products are Fe, Na, and O. The XRD analysis further confirms that NaFeO_2_ is the dominant corrosion product. A noticeable enrichment of Cr and Al is observed at the interface between the corrosion layer and the substrate. Additionally, several darker regions are present on the inner side of the corrosion layer, displaying features consistent with those from the previous experimental group. These regions are believed to result from molten salt infiltration through micro-cracks or pores within the corrosion layer.

**Figure 12:**
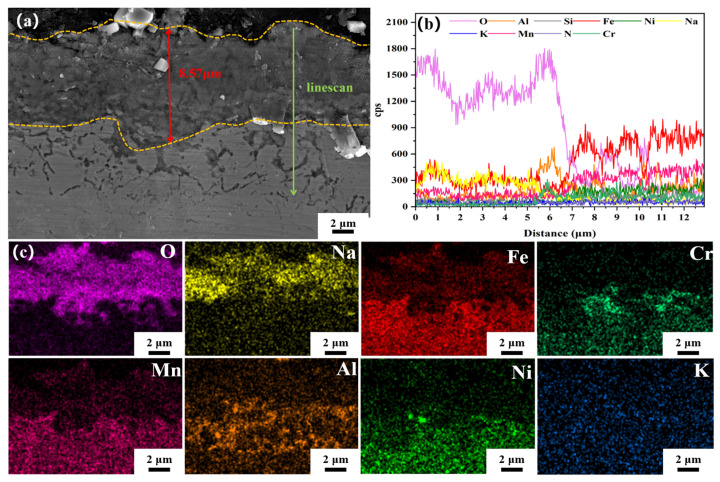
(**a**) SEM images of the cross-section of the corrosion layer of CR-Solution sample after 480 h of corrosion in molten salt, (**b**) EDS line scan analysis, (**c**) EDS area scan analysis.

Figure 12 presents the cross-sectional morphology and elemental distribution of the corrosion layer formed on the surface of CR-Solution sample after 480 h of corrosion in molten nitrate salts fusion at 1050 °C, as shown in Figure 12. The corrosion layer displays a loose and porous structure, with a thickness smaller than that observed in the annealed stainless steel sheet samples. Based on the results of EDS and XRD, the surface corrosion products are primarily identified as NaFeO_2_, and the corrosion layer has a thickness of 8.57 μm (± 0.10 μm). Surface scanning results indicate minor diffusion of Ni and Mn elements within the corrosion layer, while Cr and Al elements are enriched at the interface between the corrosion layer and the substrate, forming bright spots in this region. It is hypothesized that this zone corresponds to a Cr_2_O_3_-Al_2_O_3_ composite oxide layer.

**Figure 13:**
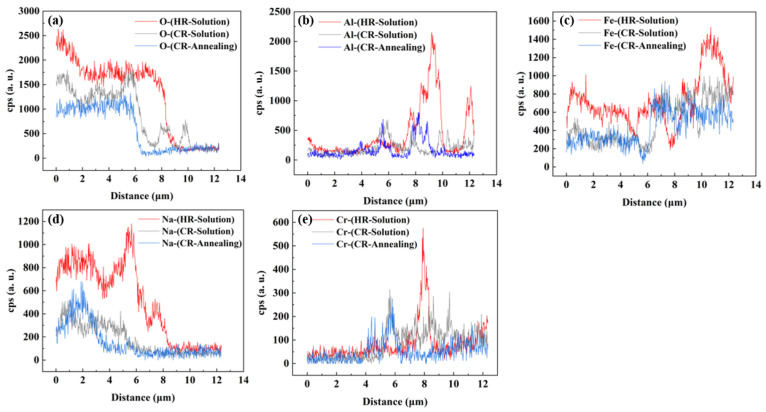
The EDS line scan maps of (**a**) O, (**b**) Al, (**c**) Fe, (**d**) Na, and (**e**) Cr in the HR-Solution, CR-Solution, and CR-Annealing conditions.

As shown in Figure 13, O, Fe, and Na elements were detected within an 8 μm depth. Comparing the O element, it was found that the diffraction peaks of the HR-Solution sample were significantly higher than those of the cold-rolled sheets treated with solid solution and annealing. The CPS peak intensities of the three types decreased in order. Additionally, when comparing the Fe and Na elements, the CPS peaks of the HR-Solution were significantly higher than those of the two cold-rolled sheets. However, the corrosion products obtained from all three samples were NaFeO_2_. Additionally, at the 8 μm to 10 μm depth range of the HR-Solution, both Al and Cr elements showed significant enrichment. Furthermore, the simultaneous presence of O, Cr, and Al elements at the 8 μm range confirms the conclusion of a composite film of Cr_2_O_3_ and Al_2_O_3_. In the two cold-rolled sheets, slight enrichment of Cr and Al elements can be observed. However, the diffraction peaks of Al and Cr enrichment in the solid CR-Solution are consistently higher than those in the annealed cold-rolled sheet. Therefore, the corrosion rates of the three samples are ranked as follows: CR-Annealing > CR-Solution > HR-Solution.

## 4. Discussion

### 4.1. The Influence of Different Processes on the Microstructure and Corrosion Performance

Among the three samples, heat treatment and rolling processing do not affect the chemical mechanism of corrosion. The three heat treatment methods only influence the thickness of the corrosion layer and the thickness of the formed composite oxide film (Al_2_O_3_-Cr_2_O_3_). During annealing, due to the influence of the precipitated phase, an effective A_l2_O_3_-Cr_2_O_3_ oxide film is not formed, but it does not mean that no Al_2_O_3_-Cr_2_O_3_ oxide film is formed. Recrystallization growth of fine grains after solution treatment: High-temperature heat treatment (1050 °C) significantly enhances the corrosion resistance and pitting resistance of the material, primarily due to the recrystallization of grains under elevated temperatures. During this process, microstructural defects introduced during deformation are effectively eliminated, and the resulting passive film on the material surface exhibits improved stability and uniformity [41]. Following solution treatment at 1050 °C, the Al, Cr, and Ni elements in High-Al 304SS are fully dissolved, leading to reduced lattice distortion, a more uniform grain size, and the absence of segregation. The homogeneous distribution of alloying elements facilitates the formation of a continuous and dense Al_2_O_3_-Cr_2_O_3_ composite oxide layer on the surface. Al preferentially oxidizes to form a compact Al_2_O_3_ protective film, which effectively inhibits the penetration of ${\mathrm{NO}}_{3}^{-}$ and O^2−^ ions from molten salt into the substrate. Furthermore, the absence of intergranular precipitates after solution treatment prevents the formation of Cr- and Al-depleted regions, thereby hindering preferential corrosion along grain boundaries [42,43,44], In addition, the electrochemical microcell effect is diminished, and the corrosion current density is reduced, both of which collectively enhance the material’s corrosion resistance. The high-temperature heat treatment effectively relieves and reduces residual stresses introduced during the rolling process. If left unaddressed, such residual stresses can exacerbate intergranular corrosion. Excessive cold deformation tends to generate a significant number of dislocations, microcracks, and grain boundary fractures within the microstructure. If these defects are not effectively eliminated, they can adversely affect intergranular corrosion behavior. Solution treatment not only reduces internal residual stresses but also promotes a more uniform grain structure, thereby improving the material’s corrosion resistance. The electrochemical microcell effect is a critical factor influencing corrosion behavior. Furthermore, the material’s microstructure plays a decisive role in determining its overall corrosion resistance. Effective elimination of structural defects is therefore essential to mitigate intergranular corrosion and enhance material performance. A well-optimized microstructure can efficiently relieve stress concentrations, thereby reducing the likelihood of localized corrosion. The synergistic effect of these improvements leads to a substantial enhancement in the material’s corrosion resistance.

**Figure 14:**
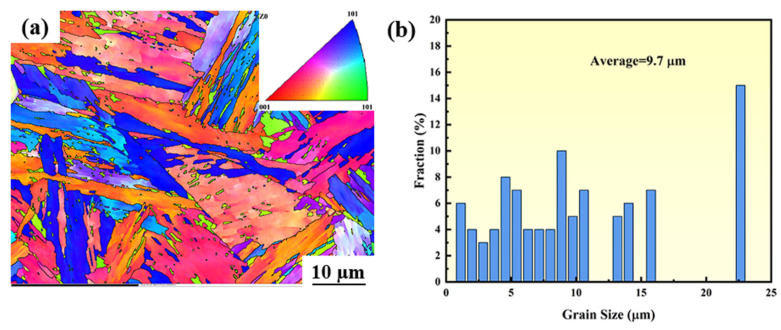
(**a**) Grain orientation distribution map (OIM) and (**b**) grain size distribution of HR-Solution.

**Figure 15:**
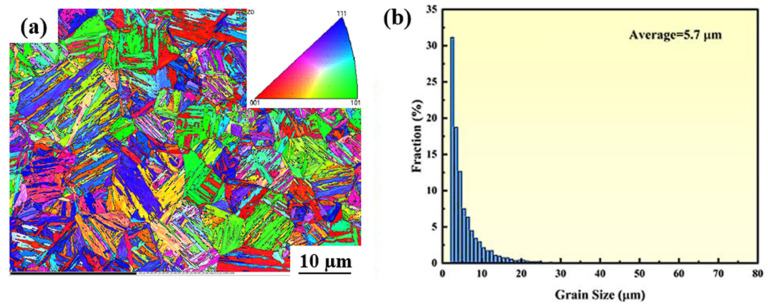
(**a**) Grain orientation distribution map (OIM) and (**b**) grain size distribution of CR-Solution sample.

The grain boundary state distribution and orientation difference statistics for HR-Solution and CR-Solution materials are presented in Figure 14 and Figure 15, respectively. In Figure 14, green lines indicate small-angle grain boundaries with an orientation difference between 2° and 10°, while black lines represent large-angle grain boundaries with an orientation difference greater than 10°. The material is predominantly composed of small-angle grain boundaries, which account for 61.17%. In Figure 15, red lines correspond to small-angle grain boundaries (2–10°), and black lines correspond to large-angle grain boundaries (>10°). The results reveal a distinct preference in grain boundary composition, with small-angle grain boundaries being dominant, accounting for as much as 79.24%. Compared to cold-rolled sheets, hot-rolled sheets exhibit coarser initial grains and fewer grain boundaries. In contrast, the cold-rolling process significantly elongates ferrite and austenite grains, forming a fibrous microstructure and generating numerous crystallographic defects such as dislocations and twins. This leads to an increased number of grain boundaries and a more complex microstructural configuration [45]. During solution treatment, alloying elements in the hot-rolled plate are more uniformly dissolved into ferrite and austenite, resulting in a homogeneous solid solution. Following the cooling stage, the microstructure remains predominantly in the solution-treated state, with minimal precipitates at grain boundaries and a well-uniformed surface passivation film. However, an imbalanced ferrite-to-austenite ratio leads to an uneven distribution of corrosion-resistant elements between the two phases, thereby compromising the material’s overall corrosion resistance.

**Figure 16:**
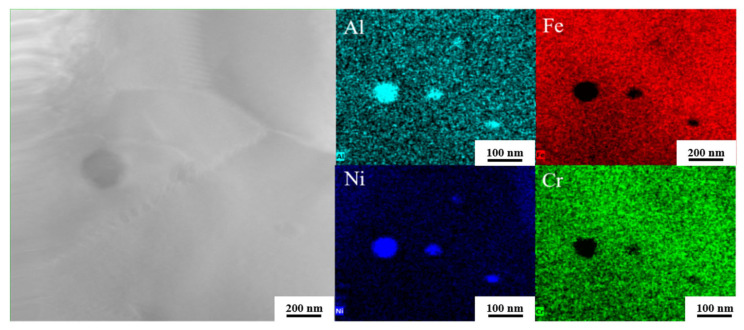
STEM images and EDS elemental distribution maps of CR-Annealing sample.

After CR-annealing in Figure 16, the amount of precipitated phases at the grain boundaries increases, including NiAl-type compounds. These phases deplete Cr at the grain boundaries, leading to the formation of localized Cr-poor regions. Concurrently, the formation of Fe_3_Al intermetallic compounds during annealing induces aluminum segregation, resulting in the development of brittle phases near the grain boundaries and exacerbating Al-poor regions. Furthermore, Cr and Al atoms diffuse toward the grain boundaries during annealing, reducing their concentrations within the grains and creating a significant compositional disparity between the grain boundaries and the grain interiors. The presence of these Cr-poor and Al-poor regions at the grain boundaries impedes the formation of a continuous Al_2_O_3_-Cr_2_O_3_ composite oxide film, thereby compromising the material’s corrosion resistance [46]. During annealing, the formation of precipitated phases accelerates the corrosion rate. The difference in thermal expansion coefficients between these phases and the matrix makes the oxide film susceptible to cracking and spalling under high-temperature conditions. Cracks propagate continuously along grain boundaries, significantly degrading the mechanical properties of the material in high-temperature molten salt environments and leading to rapid structural failure. Furthermore, microgalvanic coupling between precipitated phases and Cr-depleted regions increases the local corrosion current density, thereby intensifying localized corrosion. Cold-rolled thin plates exhibit pronounced thermal expansion anisotropy, which can lead to significant interfacial stress between the oxide film and the substrate, further promoting oxide layer spallation. In addition, high-temperature annealing reduces the austenite content in cold-rolled plates compared to hot-rolled and solution-treated counterparts, while cold rolling induces martensitic transformation. Martensite, characterized by a high density of dislocations and deformation twins, is prone to stress concentration, which facilitates microcrack initiation. These microcracks subsequently propagate along martensitic regions during corrosion, ultimately accelerating material degradation. Although partial recovery of martensite occurs during annealing, residual martensite still significantly enhances the corrosion rate of the annealed thin plates [47,48,49,50].

### 4.2. Corrosion Mechanism of High-Al 304SS in Binary Molten Nitrate Salts

**Figure 17:**
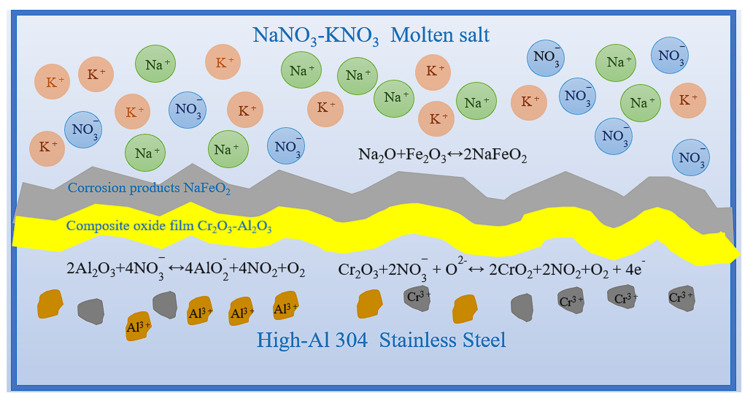
Corrosion mechanism diagram of High-Al 304SS.

The corrosion process of High-Al 304SS in binary nitrates is a complex dynamic multi-stage coupled process involving the formation of an oxide film, molten salt erosion, selective oxidation of elements, and other processes. Existing research generally believes that the corrosion process of alloys in molten salts involves not only oxidation reactions but also electrochemical reactions, including chemical reactions at the interface between the molten salt and the matrix and the dissolution of molten salt on the oxide film. In Figure 17, Sodium nitrate molten salt decomposes into sodium ions and nitrate ions (Formula (3)) when heated, and ultimately decomposes into ${\mathrm{NO}}_{2}^{-}$ and O^2−^ (Formula (4)). According to Lewis acid-base theory, acids are acceptors of oxygen ions, while bases are donors of oxygen ions. Therefore, when determining the acidity or alkalinity of molten salt fluids, the concentrations of ${\mathrm{NO}}_{2}^{-}$ and O^2−^ can be used as indicators, where O^2−^ represents alkalinity and ${\mathrm{NO}}_{2}^{-}$ represents acidity. Based on the concentration of O^2−^, the acidity or alkalinity of the molten salt fluid can be determined. When the O^2−^ concentration is low, the molten salt fluid exhibits acidic properties. At this point, the metal oxide layer formed on the surface of the metal structural material typically undergoes an acidic dissolution reaction, producing metal ions and O^2−^ (Formula (5)). When the O^2−^ concentration is high, the molten salt fluid becomes alkaline, and metal oxides generally form on the material surface in a relatively stable manner. However, when there is an excess of O^2−^, the metal oxide layer may dissolve, generating metal oxide anions (Formula (6)) [51].

(3) $${\mathrm{NaNO}}_{3}↔Na^{+}+NO_{3}^{-}$$

(4) $$NO_{3}^{-}+2e^{-}↔NO_{2}^{-}+O^{2-}$$

(5) $$M_{x}O_{y}↔xM^{z+}+yO^{2-}$$

(6) $$M_{x}O_{y}+zO^{2-}↔M_{x}O_{y+z}^{2-}$$

After corrosion in a binary molten nitrate salts at 600 °C, High-Al 304SS thin plates treated using two different oxygen showed. From the corrosion cross-sectional morphology diagrams and EDS results, it is clearly observed that the corrosion products are primarily composed of Fe, Na, and O elements. Combined with XRD results, it can be determined that the corrosion products are primarily NaFeO_2_ (Formula (7)). Regarding the formation process of this product, when the temperature of the binary molten nitrate salts exceeds 600 °C, the decomposition reaction of nitrates to nitrites in Equation (2) becomes increasingly intense. This change causes the concentration of O^2−^ in the molten salt fluid to increase, and In the initial stages of corrosion, the Fe formed on the surface of the sample undergoes complex oxidation to become Fe_3_O_4_ (Formulas (8) and (9)), which ultimately transforms into NaFeO_2_ [52] (Formulas (10) and (11).

(7) $$2\mathrm{NaN}O_{2}↔Na_{2}O+\mathrm{NO}+NO_{2}$$

(8) $$Fe^{2+}+O^{2-}↔\mathrm{FeO}$$

(9) $$3\mathrm{FeO}+O^{2-}↔Fe_{3}O_{4}+2e^{-}$$

(10) $$2Fe_{3}O_{4}+O^{2-}↔3Fe_{2}O_{3}+2e^{-}$$

(11) $$Fe_{2}O_{3}+Na_{2}O↔2\mathrm{NaFe}O_{2}$$

In high-temperature environments, Al elements oxidize more readily than Cr and Fe elements due to their higher oxidation drive, forming an Al_2_O_3_ oxide film (Formula (12)). At this point, the dense oxide film can effectively prevent molten salt from penetrating and corroding the matrix. However, ${\mathrm{NO}}_{3}^{-}$ in molten salt decomposes at high temperatures to produce O^2−^ and ${\mathrm{NO}}_{3}^{-}$, which react with Al_2_O_3_ to form soluble aluminum salts, causing the Al_2_O_3_ film to dissolve locally (Formula (13)), forming holes and exposing the Fe and Cr matrix.

2Al + 3O^2−^ ↔ Al_2_O_3_ + 6e^−^ (12)

(13) $$2{\mathrm{Al}}_{2}O_{3}+4NO_{3}^{-}↔4{\mathrm{AlO}}_{2}^{-}+4{\mathrm{NO}}_{2}+O_{2}$$

In addition, Cr is also present in the corrosion products in the form of compounds. First, Cr is oxidized to form Cr_2_O_3_ (Formula (14)). When the concentration of nitrate ions in the binary nitrate molten salt is high and the temperature is high, Cr^3+^ is further oxidized to Cr^4+^, thereby forming CrO_2_ (Formula (15)), as shown in the reaction equation below.

2Cr + 3O^2−^ ↔ Cr_2_O_3_ + 6e^−^ (14)

(15) $${\mathrm{Cr}}_{2}O_{3}+O^{2-}+2{\mathrm{NO}}_{3}^{-}↔2{\mathrm{CrO}}_{2}+2{\mathrm{NO}}_{2}+O_{2}+4e^{-}$$

From the above reactions, it can be seen that the main corrosion product generated is NaFeO_2_. This is because when the corrosion temperature exceeds 600 °C, the concentration of O^2−^ in the molten salt increases, causing the alkalinity of the molten salt to increase, and some of the oxides produced in the initial stage of corrosion are converted into Na-containing compounds.

### 4.3. Comparative Analysis of Corrosion Rates for Various Alloys Across Diverse Environmental Conditions

**Figure 18:**
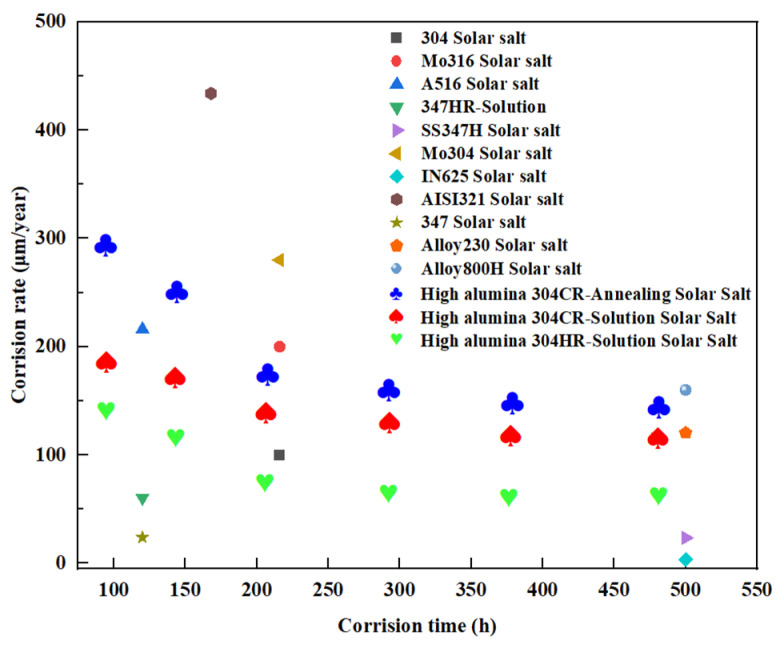
Corrosion rates of different alloys in molten salts.

Figure 18 shows the corrosion rates of the alloy as reported in the relevant literature [53,54,55,56,57,58,59,60,61,62] and three selected groups of High-Al 304SS specimens in this study, as indicated by the enlarged blue, red and green marks. During the corrosion testing conducted over 480 h, the HR-Solution exhibited favorable corrosion resistance, characterized by relatively low corrosion rates. In the binary molten nitrate salts environment at 565 °C, the corrosion rates of two types of High-Al 304SS cold-rolled plates subjected to different heat treatments were slightly higher than that of the 347H heat-resistant steel but lower compared to other alloys. The incorporation of aluminum facilitates the formation of a stable and protective passive film, thereby enhancing the material’s corrosion resistance. The Al_2_O_3_ and Cr_2_O_3_ oxide layers jointly form a “barrier-type” composite protective film, which effectively inhibits the migration of corrosive ions in the molten salt. Solid solution treatment improves the corrosion resistance of High-Al 304SS by suppressing phase precipitation and promoting microstructural homogenization. However, annealing treatment leads to the formation of grain boundary precipitation phases and Al/Cr depletion zones, and martensite is not fully eliminated even after high-temperature annealing. These factors can collectively contribute to a decrease in molten salt corrosion resistance. High-Al 304SS hot-rolled sheets subjected to solid solution treatment exhibit excellent corrosion resistance in molten nitrate salts environments, and their high-temperature mechanical properties generally meet the requirements for molten salt storage tank materials. Therefore, they have the potential to serve as a cost-effective alternative to 347H heat-resistant steel in molten salt storage applications. However, the corrosion resistance of High-Al 304SS cold-rolled sheets subjected to solid solution treatment is diminished, as cold-rolling disrupts the balance between ferrite and austenite phases, resulting in an uneven distribution of corrosion-resistant elements and consequently failing to meet the requirements for absorbers. Nevertheless, the corrosion rate of these sheets can be reduced by extending the solid solution treatment time or increasing the treatment temperature.

## 5. Conclusions

To reduce the cost of concentrated solar power (CSP) systems, this study systematically investigated the corrosion behavior of High-Al 304SS in NaNO_3_-KNO_3_ molten salt. The main conclusions are as follows: The corrosion resistance ranking was: HR-Solution > CR-Solution > CR-Annealing. HR-Solution sample exhibited the optimal performance with a corrosion rate of only 64.43 μm/year. The corrosion resistance of the materials exhibits a positive correlation with the density of the composite oxide film. The HR-Solution formed a dense and continuous Al_2_O_3_-Cr_2_O_3_ oxide film, while the CR-Solution demonstrated intermediate oxide film density. In contrast, the CR-Annealing showed reduced oxide film density due to the precipitation of NiAl phases during the annealing process. The high defect density and grain boundary energy introduced by cold rolling accelerated corrosion in CR-Solution. In CR-Annealing, the precipitation of NiAl phases and Cr segregation further degraded corrosion resistance. In contrast, HR-Solution, which underwent no cold rolling, demonstrated uniform distribution of Al/Cr/Ni elements, thereby reducing segregation and localized corrosion. HR-Solution can serve as an ideal low-cost alternative to 347H stainless steel for molten salt storage tanks.

## Figures and Tables

**Table 1:** Composition of 304 SS and High-Al 304SS.

Ingredients	Element Content (wt.%)								
	C	Si	P	S	Al	Mn	Ni	Cr	Fe
304SS	≤0.08	≤1.00	≤0.045	≤1.00	/	≤2.00	8.00	18.0	Basal
High-Al 304SS	0.04	0.72	<0.035	<0.03	2.88	1.9	8.19	12.71	Basal

**Table 2:** Quantitative analysis data of HR-Solution sample based on the XRD results.

Name	K Value	Weight wt.%
NaFeO_2_	3.94	54.00
Mn_3_O_4_	2.64	19.60
NiCr_2_O_4_	4.60	13.30
CrO_2_	3.92	5.10
CrO_3_	3.18	8.00

**Table 3:** Surface EDS elemental analysis of HR-Solution sample after exposure to molten salt for different durations.

Location	Element Content (At.%)									
	N	O	Na	Al	Si	K	Cr	Mn	Fe	Ni
Point 1	0	53.36	23.58	1.03	0.19	0.23	0	0	21.61	0
Point 2	0.97	24.27	2.57	15.16	1.50	0.22	3.03	0	41.86	10.42
Point 3	0.50	62.79	12.52	2.08	0.80	0.43	0	0	20.88	0
Point 4	0	52.86	23.24	1.53	0.16	0.08	0	0	22.13	0
Point 5	0.29	52.74	27.47	1.52	0.50	0.30	0	0	16.91	0.27
Point 6	0	57.50	17.95	0.16	0.01	0.47	0	0	23.91	0

**Table 4:** Surface EDS elemental analysis of CR-Solution sample after exposure to molten salt for different durations.

Location	Element Content (At.%)									
	N	O	Na	Al	Si	K	Cr	Mn	Fe	Ni
Point 1	3.89	52.59	20.9	1.80	0.75	2.16	0	0	17.91	0
Point 2	7.38	14.90	1.92	11.28	1.40	0.21	2.53	0	52.46	7.92
Point 3	0.26	60.65	4.00	1.01	0.47	0.30	0	0	33.31	0
Point 4	0	51.46	24.72	0.62	0.06	0.04	0	0	23.10	0
Point 5	0	52.97	21.98	1.07	0.17	0.06	0	0	23.75	0
Point 6	0	64.62	19.26	0.12	0.07	0.16	0	0	15.77	0

**Table 5:** Surface EDS elemental analysis of CR-Annealing sample after exposure to molten salt for different durations.

Location	Element Content (At.%)									
	N	O	Na	Al	Si	K	Cr	Mn	Fe	Ni
Point 1	0	57.80	9.90	1.66	1.30	0.04	0	0	29.30	0
Point 2	0.42	10.68	0.83	6.69	1.13	0.09	9.14	0.10	66.16	4.76
Point 3	0	61.58	12.90	5.14	2.78	0.49	0	0	17.11	0
Point 4	0	50.12	23.10	1.29	0.15	0.05	0	0	25.29	0
Point 5	0	51.99	26.79	0.45	0.20	0.24	0	0	20.33	0
Point 6	0	56.28	32.81	0.08	0.08	0.04	0	0	10.71	0
Point 7	0.55	61.84	14.92	0.25	0.14	0.18	0	0	22.21	0

## Data Availability

The original contributions presented in this study are included in the article/Supplementary Material. Further inquiries can be directed to the corresponding authors.

## Supplementary Materials

The following supporting information can be downloaded at: https://www.mdpi.com/article/10.3390/ma18194513/s1, Figure S1: OM images of the surface morphology of CR-Annealing at 800 °C. Figure S2: XRD patterns of High-Al 304SS samples. Figure S3: The cross-section SEM images of CR-Annealing sample before corrosion. Figure S4: The cross-section SEM images of CR-Solution sample before corrosion. Figure S5: The cross-section SEM images of HR-Solution sample before corrosion.
