# Supplementary material for: Differential Corrosion Behavior of High-Aluminum 304 Stainless Steel in Molten Nitrate Salts: The Roles of Rolling and Heat Treatment
Source: Materials (Basel). 2025 Sep 28;18(19):4513. doi: 10.3390/ma18194513 (PMC12525352; doi:10.3390/ma18194513)
Supplement: Supplementary file 1 [file materials-18-04513-s001.zip › materials-3836599-supplementary.pdf]

materials

MDPI

Supporting Information

Differential Corrosion Behavior of High-Aluminum 304 Stainless Steel in Molten Nitrate Salts: The Roles of Rolling and Heat Treatment

Weijie Tang [1], Kan Zhou [1], Zhenguo Li [2], Lifu Xin [2], Dexian Huang [3], Faqi Zhan [1,*], Penghui Yang [1], Haicun Yu [1] and Peiqing La [1,*]

[1] State Key Laboratory of Advanced Processing and Recycling of Non-Ferrous Metals, School of Materials Science and Engineering, Lanzhou University of Technology, Lanzhou 730050, China; tangweijie1116@gmail.com (W.T.); 18215161072@163.com (K.Z.); yangph@lut.edu.cn (P.Y.); yuhcyu@lut.edu.cn (H.Y.)

[2] Sun Sum Technology Co. Ltd., Beijing 100020, China; lizhenguo@nmpower.com.cn (Z.L.); xinlifu@nmpower.com.cn (L.X.)

[3] Sun Sum (Gansu) Technology Co. Ltd., Lanzhou 730000, China; huangdexian@nmpower.com.cn

* Correspondence: zhanfaqi@lut.edu.cn (F.Z.); pqla@lut.edu.cn (P.L.)

Figure Captions

Figure S1. OM images of the surface morphology of CR-Annealing at 800 °C.

Figure S2. XRD patterns of High-Al 304SS samples.

Figure S3. The cross-section SEM images of CR-Annealing sample before corrosion.

Figure S4. The cross-section SEM images of CR-Solution sample before corrosion

Figure S5. The cross-section SEM images of HR-Solution sample before corrosion.

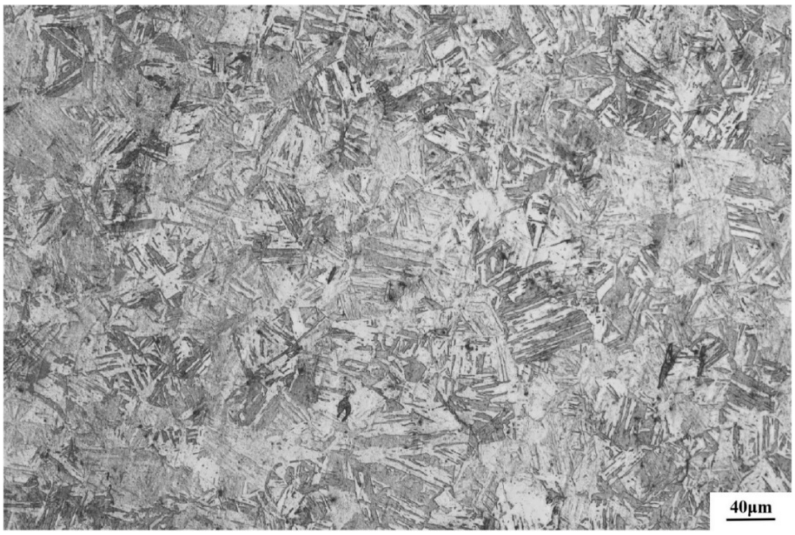

Figure S1. OM images of the surface morphology of CR-Annealing sample at 800 °C.

 https://doi.org/10.3390/ma18194513

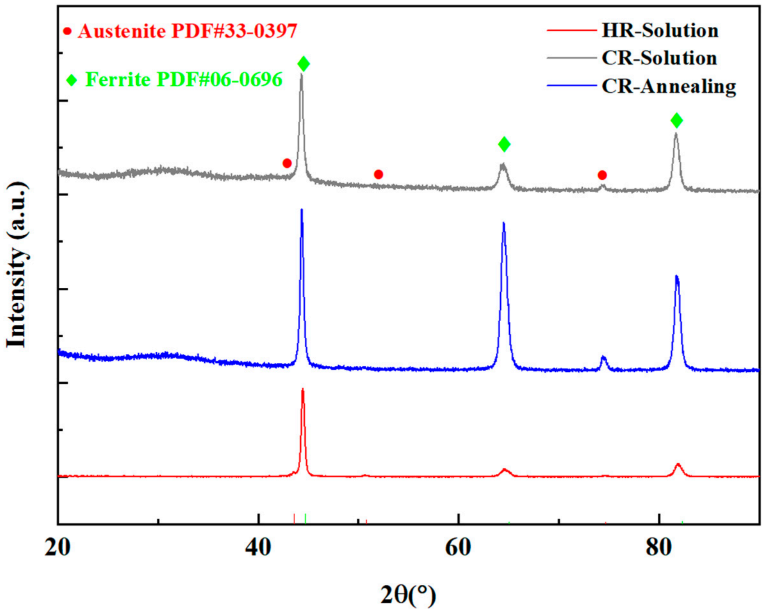

Figure S2. XRD patterns of High-Al 304SS samples.

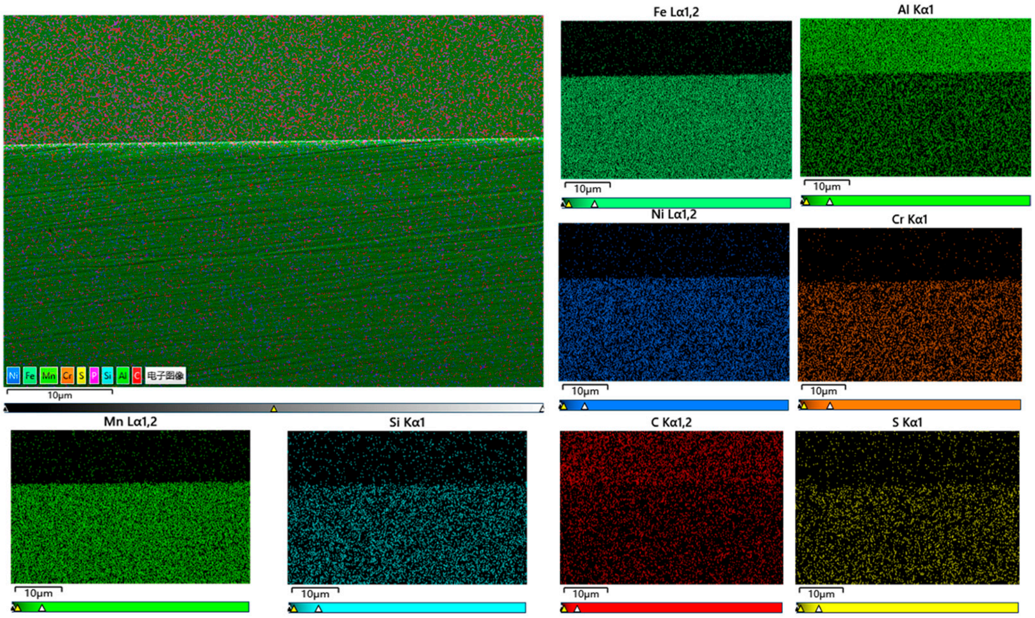

Figure S3. The cross-section SEM images of CR-Annealing sample before corrosion.

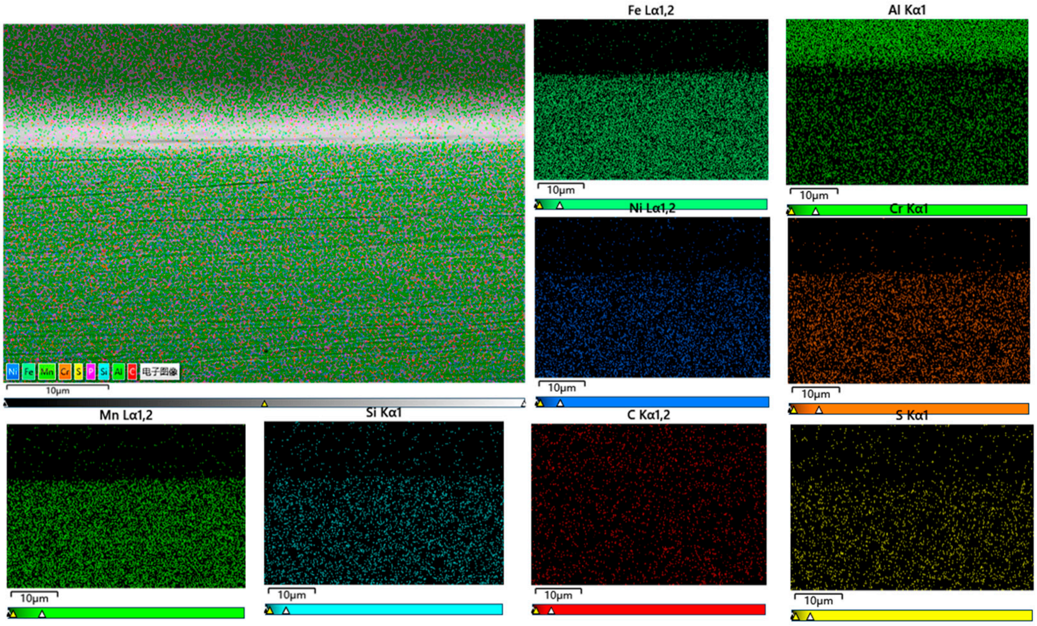

Figure S4. The cross-section SEM images of CR- Solution sample before corrosion.

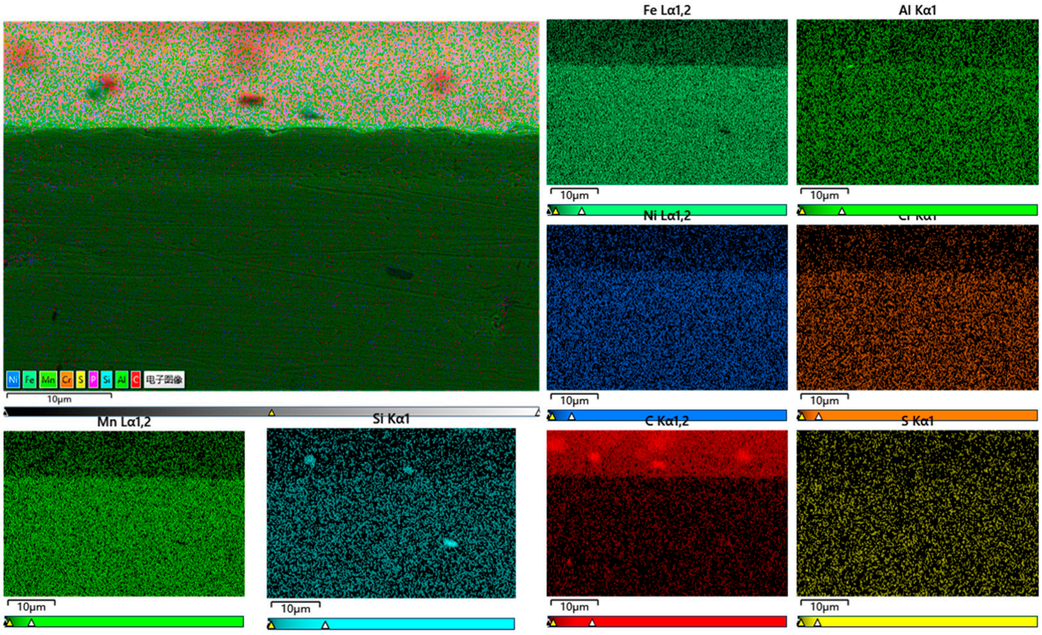

Figure S5. The cross-section SEM images of HR-Solution sample before corrosion.
